# BMSCs-assisted injectable Col I hydrogel-regenerated cartilage defect by reconstructing superficial and calcified cartilage

**DOI:** 10.1093/rb/rbz028

**Published:** 2019-11-22

**Authors:** Hanxu Cai, Peilei Wang, Yang Xu, Ya Yao, Jia Liu, Tao Li, Yong Sun, Jie Liang, Yujiang Fan, Xingdong Zhang

**Affiliations:** 1 National Engineering Research Center for Biomaterials, Sichuan University, 29 Wangjiang Road, Chengdu 610064, P. R. China; 2 Department of Gynecology and Obstetrics, Development and Related Disease of Women and Children Key Laboratory of Sichuan Province, Key Laboratory of Birth Defects and Related Diseases of Women and Children, Ministry of Education, West China Second Hospital, Sichuan University, 20 Renmin South Road, Chengdu 610041, P. R. China

**Keywords:** BMSCs, injectable Col I hydrogel, superficial cartilage, calcified cartilage

## Abstract

The self-healing capacity of cartilage was limited due to absence of vascular, nervous and lymphatic systems. Although many clinical treatments have been used in cartilage defect repair and shown a promising repair result in short term, however, regeneration of complete zonal structure with physiological function, reconstruction cartilage homeostasis and maintaining long-term repair was still an unbridgeable chasm. Cartilage has complex zonal structure and multiple physiological functions, especially, superficial and calcified cartilage played an important role in keeping homeostasis. To address this hurdle of regenerating superficial and calcified cartilage, injectable tissue-induced type I collagen (Col I) hydrogel-encapsulated BMSCs was chosen to repair cartilage damage. After 1 month implantation, the results demonstrated that Col I gel was able to induce BMSCs differentiation into chondrocytes, and formed hyaline-like cartilage and the superficial layer with lubrication function. After 3 months post-surgery, chondrocytes at the bottom of the cartilage layer would undergo hypertrophy and promote the regeneration of calcified cartilage. Six months later, a continuous anatomical tidemark and complete calcified interface were restored. The regeneration of neo-hyaline cartilage was similar with adjacent normal tissue on the thickness of the cartilage, matrix secretion, collagen type and arrangement. Complete multilayer zonal structure with physiological function remodeling indicated that BMSCs-assisted injectable Col I hydrogel could reconstruct cartilage homeostasis and maintain long-term therapeutic effect.

## Introduction

Cartilage defect caused by abrasion with the aging of population or traumatic injury was an urgent problem at the clinic due to their limited potential for self-healing [1, 2]. Current clinical techniques for treating cartilage defect, including microfracture, autologous/allogeneic implantation, were palliative treatment methods and not able to guarantee long-term curative effect [1–3]. The restoration of cartilage homeostasis played an important role in maintaining long-term repair efficacy. It is depended on complete and functional hierarchical structure and function reconstruction [4–7]. According to distinguished cell subpopulations with different morphology, density, arrangement and gene expression, cartilage was clearly divided into three zones including the superficial, intermediate and calcified zones [4, 5, 8]. The superficial zone had the flattened chondrocytes, progenitor cells (PCs) and joint lubricants proteoglycan 4 (Prg 4) [6, 7, 9–12]. PCs would endow the cartilage with a certain capacity for self-renewal under mechanical abrasion [6, 7]. Meanwhile, Prg 4 would decrease chondrocytes apoptosis and invent the process of cartilage deterioration by reducing the friction coefficient of joint surface [10, 11]. The intermediate zone contained round chondrocytes and had the highest content of GAGs and Col II to support compressive and tensile strength [4, 5, 7]. The calcified cartilage as a barrier between the avascular cartilage and the hypervascularized subchondral bone prevented the mineralization and vascularization of hyaline cartilage [13, 14]. The superficial zone and the bottom calcified cartilage were considered as key structures in regulating cartilage homeostasis [9, 15]. However, the regeneration of the two functional structures remained a considerable challenge in clinic.

With the technique advance over recent decades, current strategies have focused on tissue engineering technology. Cells, signals and scaffolds were the major elements in tissue engineering [1]. Chondrocytes as the main cell type in cartilage tissue was an ideal cell source for cartilage tissue engineering. Nevertheless, chondrocytes using in clinical treatment had some limitations such as difficult to extract, low harvesting cell numbers and easily losing their phenotype during the culture and expansion process [4, 16, 17]. Stem cells have been considered as a more ideal cell source in tissue engineering. They were comparatively easy to isolate and proliferate [1]. However, the main challenge of using MSCs for cartilage regeneration was how to induce the chondrogenic differentiation of MSCs with multipotent characteristic [1, 4, 15]. Many studies incorporated growth factors to regulate MSCs differentiation and promote cartilage repair [4, 18]. Several problems limited the clinical application of growth factors [19]. The concentration of growth factors was great importance for cartilage regeneration, in appropriate using might cause a negative impact on curative effect [20]. High cost of goods, difficult to assurance quality, manufacturing and storage also limited the application of growth factors [21]. Therefore, new strategies need be explored.

Collagen hydrogels and collagen-based hydrogels have been used in cartilage defect repair [22, 23]. Collagen was one of the most abundant proteins in animal and had good biocompatibility [24]. Previous researches were always combined collagen with growth factors to promote cartilage regeneration [25, 26]. The limitations and risks of using growth factors have been introduced. In recent years, some studies found Col I hydrogel without addition of any exogenous growth factor or other media could induce the chondrogenic of BMSCs [27, 28]. The results might be ascribed to Col I hydrogel scaffold provide a suitable microenvironment and aggregate the signal molecule for chondrogenesis [27, 28]. Previous studies have confirmed that type I collagen could induce BMSCs to differentiate into chondrocytes *in vitro* and rabbits subcutaneous, but the curative effect on regenerating cartilage tissue, reconstructing superficial and calcified cartilage has not yet been verified. In this study, injectable Col I hydrogel was used to provide suitable physiological microenvironment for the chondrogenic of BMSCs, superficial and calcified cartilage reconstruction and homeostasis remodeling. During surgery, the injectable scaffolds *in situ* could form any anticipated shapes, match irregular defects and improve the integration between the regenerated tissue and the host tissue via a minimally invasive method [29–31]. The results of regenerated cartilage tissue showed that BMSCs-assisted injectable Col I hydrogel scaffold helped to regenerate complete hierarchical cartilage structure with corresponding biosynthetic function, remold the homeostasis of cartilage for long-term curative effect.

## Materials and methods

### Isolation and culture of BMSCs

All animal experiments were approved by the Sichuan University Medical Ethics Committee. All procedures were operated in accordance with the guidelines for care and use of Laboratory Animals of Sichuan University. One adult male New Zealand White rabbits (about 2.5–3 kg) was euthanized by overdose of pentobarbital sodium. Bone marrows were collected from the tibial and femoral bones. Then BMSCs were isolated by gradient-centrifugation method with a kit (LGS1090, TBD). The isolated BMSCs were cultured in alpha-modified Eagle’s medium (a-MEM, Hyclone) supplemented with 20% fetal bovine serum (FBS, Gibco) and 1% penicillin–streptomycin (Hyclone). After the first passage, a-MEM was changed to add with 10% FBS.

### Preparation of type I collagen

Type I collagen (Col I) was extracted as reported previously by our research group [27, 28]. Briefly, it was extracted from calf skin and dissolved in acetic acid. Then the dissolved collagen would be depurated by sodium chloride fractionation and fibril assembly. Then the collagen solution was frozen and lyophilized. Col I hydrogel was dissolved into 0.5 mol/l acetic acid and regulated the concentration of Col I as 17 mg/ml.

### Preparation and characterization of collagen hydrogel

The pH value of the Col I solution was regulated to 7.2 by 1.0 mol/l NaOH, then PBS was added to regulate the final concentration at 10 mg/ml. The neutral Col I solution was injected into the mold and kept at 37°C for 0.5 h. The hydrogels were frozen in liquid nitrogen and immediately lyophilized. Then the hydrogels were coated with a layer of gold for observing the morphology by scanning electron microscopy (SEM, HITACHI S-800, Japan). The mechanical property of the Col I hydrogels was measured by dynamic mechanical analyzer (DMA, TA Instruments Q800, USA).

### Animal surgery

When BMSCs reached 80–90% confluence at passage two, cells would be enzymatically dissociated and collected as cell suspension. Meanwhile, the pH value of the Col I solution was regulated to 7.2 by the addition of 1.0 mol/l NaOH in ice bath, and added PBS to regulate the concentration of Col I solution at 10 mg/ml. Then the cells suspension was added and mixed with Col I solution (5 × 10^6^ cells/ml) and kept in ice bath. Total of 18 adult male New Zealand White rabbits (about 2.5–3 kg) were anaesthetized by intravenous injection pentobarbital sodium (40 mg/kg). Then the knee joints were opened and the surface of groove was exposed by laterally dislocating the patella. A cartilage defect (4 mm diameter, 2 mm depth) which was larger than the critical-size defect of cartilage self-healing was created with stainless drill [32]. Subsequently, the neutral Col I solution (10 mg/ml) mixed with BMSCs (5 × 10^6^ cells/ml) was injected into osteochondral defect, and another defect in the same rabbit was untreated (UT) as the control group (*n* = 6). After the Col I formed hydrogel, the joint capsule and skin were closed with interrupted sutures. All rabbits were sent back to their individual cages and intramuscularly administered antibiotic persistent 3 days.

### Gross morphology observe and assessment

After 1, 3 and 6 months later, rabbits were euthanized by injection overdose of pentobarbital sodium. Once the articular cavities were opened, the defect sites and surrounding tissues were examined. The joint samples were acquired and photographed by etallographic microscope (HDMI-A1, Saike Digital). Samples of each group were blindly and independently assessed by three evaluators using International Cartilage Repair Score (ICRS) system as shown in Table 1 [33].

**Table 1:** International Cartilage Repair Society (ICRS) cartilage repair assessment tool

	Criteria	Points
Degree of defect repair	Level with surrounding cartilage	4
	75% repair of defect depth	3
	50% repair of defect depth	2
	25% repair of defect depth	1
	0% repair of defect depth	0
Integration to border zone	Complete integration with surrounding cartilage	4
	Demarcating border <1 mm	3
	3/4 of graft integrated, 1/4 with a notable border >1 mm width	2
	1/2 of graft integrated with surrounding cartilage, 1/2 with a notable border >1 mm	1
	From no contact to 1/4 of graft integrated with surrounding cartilage	0
Macroscopic appearance	Intact smooth surface	4
	Fibrillated surface	3
	Small, scattered fissure or cracks	2
	Several, small or few but large fissures	1
	Total degeneration of grafted area	0
Overall score	Grade I normal	12
	Grade II nearly normal	11-8
	Grade III abnormal	7-4
	Grade IV severely abnormal	3-1

### Histological, immunohistochemical and immunofluorescent assessment

All samples were fixed in 4% paraformaldehyde for 2 weeks, decalcified in 10% EDTA for 6 weeks, dehydrated in graded alcohol, embedded in paraffin and sectioned at 5 μm thickness. Paraffin sections were stained with hematoxylin–eosin (HE), toluidine blue (TB) and Safranin-O (Saf.O) counterstained with fast green for histological evaluation, score and detection of GAGs expression. Histological scores were evaluated using a previously established scoring system for osteochondral repair, as shown in Table 2 [18, 34]. These staining results were recorded by a scanner (Pannoramic MIDI, 3D HISTECH). Picrosirius red (PSR) was stained for evaluating the type and arrangement of collagen fibers. PSR staining was recorded by polarizing microscope (NIKON).

**Table 2:** Histological scoring system for the chondral region

	Criteria	Score
Morphology of neo-formed surface tissue	Exclusively AC	4
	Mainly hyaline cartilage	3
	Fibrocartilage (spherical morphology observed with ≥75% of cells)	2
	Only fibrous tissue (spherical morphology observed <75% of cells	1
	No tissue	0
Thickness of neo-formed cartilage	Similar to the surrounding cartilage	3
	Greater than surrounding cartilage	2
	Less than the surrounding cartilage	1
	No cartilage	0
Joint surface regularity	Smooth, intact surface	3
	Surface fissures (<25% neo-surface thickness)	2
	Deep fissures (25–99% neo-surface thickness)	1
	Complete disruption of the neo-surface	0
Chondrocyte clustering	None at all	3
	<25% chondrocytes	2
	25–100% chondrocytes	1
	No chondrocytes present (no cartilage)	0
Chondrocyte and GAG content of neo-cartilage	Normal cellularity with normal Saf.O staining	3
	Normal cellularity with moderate Saf.O staining	2
	Clearly less cells with poor Saf.O staining	1
	Few cells with no or little Saf.O staining	0
Chondrocyte and GAG content of adjacent cartilage	Normal cellularity with normal GAG content	3
	Normal cellularity with moderate GAG content	2
	Clearly less cells with poor GAG content	1
	Few cells with no or little GAG or no cartilage	0

Collagen II (Col II) was detected with mouse antirabbit Col II primary antibody (NBP2-33343) by immunohistochemical (IHC) staining. Col X, Prg 4, TGF-β and BMP-2 were stained with mouse antirabbit Col X primary antibody (Thermo MA5-14268), mouse antirabbit Prg 4 primary antibody (MABT401), mouse antirabbit TGF-β1 primary antibody (Abcam, ab190503) and mouse antirabbit BMP-2 primary antibody (Abcam, ab6285) by immunofluorescent (IF) staining, respectively. The IHC/IF staining protocol was briefly described. After rehydrating the sections, they were immersed in Tris-EDTA antigen retrieval buffer (pH = 9.0) for antigen retrieval. Ten percent of goat normal serum solution was used to invent other nonspecific binding. Two  hours later of blocking, primary antibody was added onto the sections and incubated at 4°C overnight. In IHC staining, the sections were successively treated with 3% H_2_O_2_ for exhausting endogenous peroxidase, polymer helper and polyperoxidase-antimouse IgG (ZSGB-BIO, PV-9002). Finally, the sections were incubated with DAB for 10 min to visualize the staining and hematoxyl for 30 s to tag nucleus. After treating with primary antibody, for IF staining, the goat antimouse IgG (Thermo Fisher Scientific) was incubated for 1 h. Then the sections were incubated with DAPI for 5 min to tag nucleus.

### Statistics

Statistical analysis comparing different scaffolds with different months was performed by one-way ANOVA followed by *post-hoc* tests. All analyses were performed by SPSS software and the significance level was set at *P < 0.05, **P < 0.01 and ***P < 0.001.

## Results and discussion

### Characterization of the Col I hydrogel

**Figure 1:**
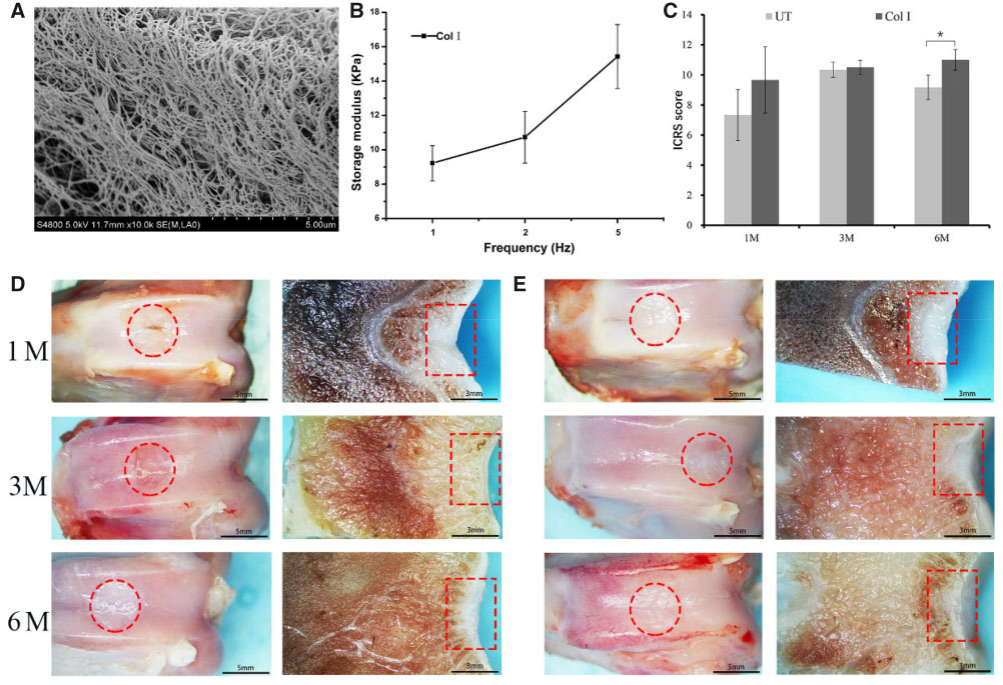
(**A**) SEM Images for the Col I hydrogel. (**B**) The mechanical property of Col I hydrogel. (**C**) ICRS macroscopic score of the regenerated tissue in different groups; macroscopic appearance of the regenerated tissue in UT group (**D**) and BMSCs-assisted Col I group (**E**) at 1, 3 and 6 months. The scale bar on the left column is 5 mm, and the right column is 3 mm

The fibers and porous in Col I hydrogel were showed in SEM (Fig. 1A). The fibers were densest and uniform in diameter. They entwined each other to form 3D networks with homogeneous pores distribution to effectively transport the nutrients and growth factors, and support cell adhesion and activity in 3D structure. Frequency sweep dynamic data of Col I hydrogel was shown in Fig. 1B. The storage modulus of the hydrogel represented the energy stored per unit strain. The higher the frequency, the bigger the storage modulus of Col I hydrogel was.

### Macroscopic assessment

The synovial fluid in all samples was found to be clear and normal. The adjacent and regenerated tissue was normally without suppuration, degeneration and other obvious abnormal conditions. There was no scaffold delaminated or migrated from the defect into the joint cavity. Then the macroscopic analysis of the defect site was evaluated (Fig. 1D and E). At 1 month post-implantation, the Col I hydrogel group had regenerated a complete, smooth and uniform cartilage-like tissue which was well integration with adjacent host cartilage. But there was a clear demarcation between the neo-tissue and the native articular cartilage. Compared with BMSCs-assisted Col I gel group, the implant sites of the UT group filled with incomplete cartilage-like tissue with some distinct fissures or cracks. After 3 months, in BMSCs-assisted Col I gel group, the neo-tissue was similar to the surrounding native tissue in appearance and the boundary between neo-tissue and adjacent normal tissue was indistinct. Although the defect sites of UT group also basically filled with neo-tissue, a clear boundary between the neo-tissue and the neighboring articular cartilage could be obviously observed due to some irregular cracks. By 6 months, the macroscopic morphology and cartilage thickness have been very similar to surrounding normal cartilage tissue in BMSCs-assisted Col I gel group. However, there was irregular and rough surface in UT group. There was a clear distinction between the neo-tissue with the native cartilage in morphology.

The results of gross morphological scores were consistent with the visual evaluation analysis (Fig. 1C). At 1 and 3 months, there was no statistically significant difference between the UT group and the BMSCs-assisted Col I gel group. After 6 months, the BMSCs-assisted Col I gel group displayed significantly higher scores when compared to the UT group (*P* < 0.05). By 6 months later, the morphological scores of the BMSCs-assisted Col I gel group was infinitely close to the maximum score (max score = 12), indicated the macroscopic morphology of the regenerated tissue in BMSCs-assisted Col I gel group was basically close to normal articular cartilage. Overall, these results demonstrated that the gross morphological appearance of the defect sites in BMSCs-assisted Col I gel group was deemed to be superior to UT group. What is more, at 6 months, the macroscopic morphology and cartilage thickness of neo-tissue were infinitely similar to adjacent host cartilage in BMSCs-assisted Col I gel hydrogel group.

### Microscopic assessment

**Figure 2:**
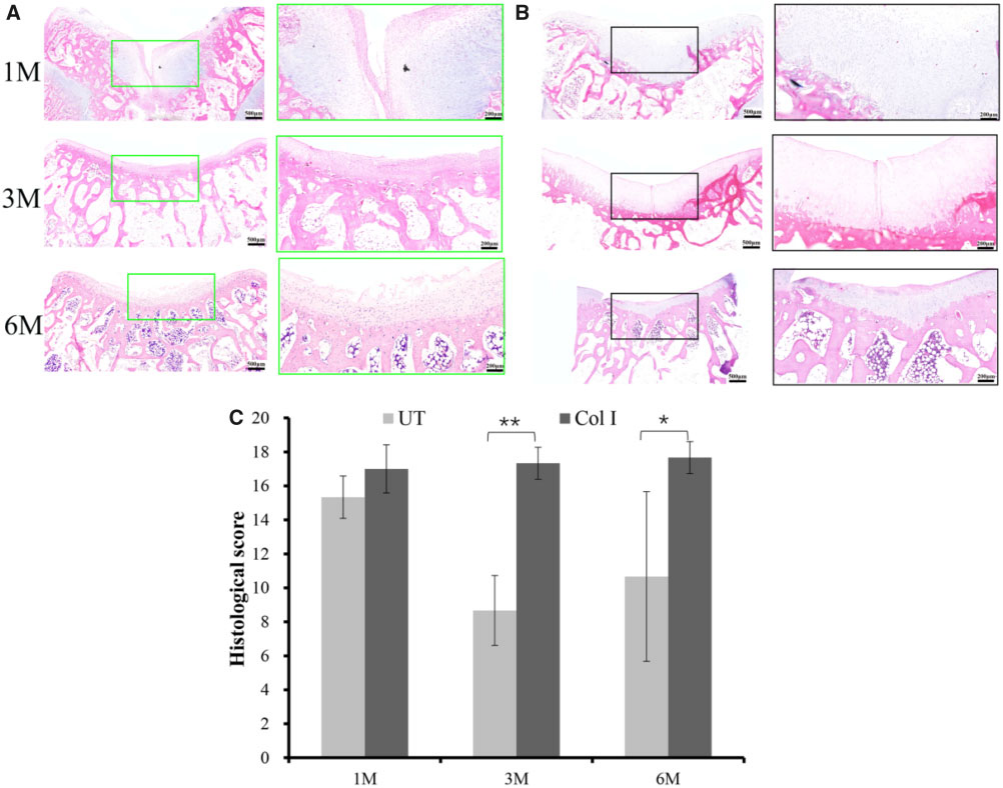
The H&E staining of the regenerated tissue in UT group (**A**) and BMSCs-assisted col I group (**B**) at 1, 3 and 6 months. The scale bar on the left column is 500 μm, and the right column is 200 μm. Histological score of the regenerated tissue in different groups (**C**)

**Figure 3:**
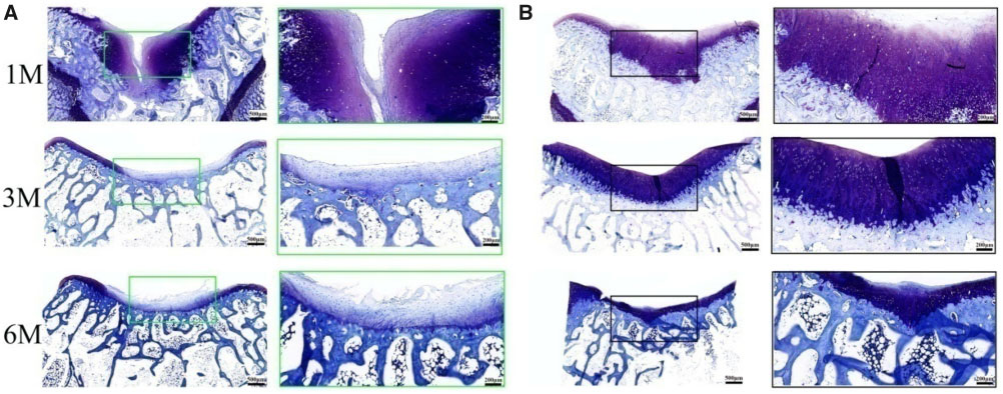
The TB staining of the regenerated tissue in UT group (**A**) and BMSCs-assisted Col I group (**B**) at 1, 3 and 6 months. The scale bar on the left column is 500 μm, and the right column is 200 μm

**Figure 4:**
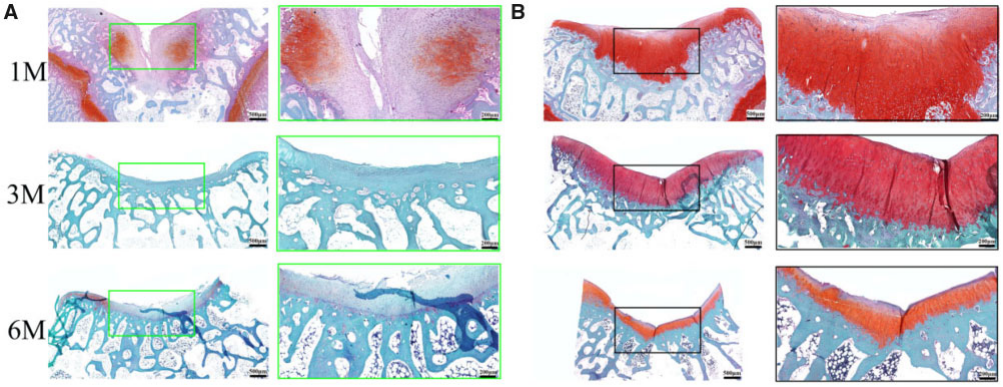
The Saf.O staining of the regenerated tissue in UT group (**A**) and BMSCs-assisted Col I group (**B**) at 1, 3 and 6 months. The scale bar on the left column is 500 μm, and the right column is 200 μm

**Figure 5:**
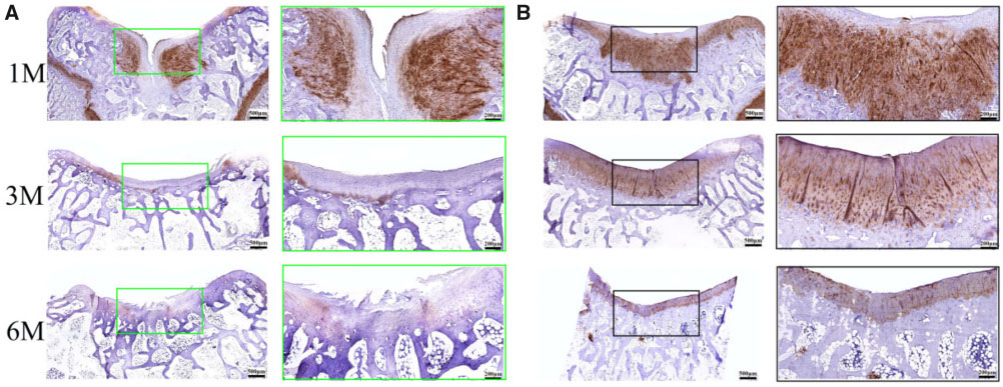
The IHC staining for Col II of the regenerated tissue in UT group (**A**) and BMSCs-assisted Col I group (**B**) at 1, 3 and 6 months. The scale bar on the left column is 500 μm, and the right column is 200 μm

Histological analysis of the cartilage defect repair supported the macroscopic findings that the BMSCs-assisted Col I gel group was able to enhance the repair of articular cartilage, relative to the UT group. At 1 month post-implantation, the BMSCs-assisted Col I gel group displayed a newly regenerated tissue that had occupied all the defect area and smoothly integrated into the surrounding healthy cartilage as demonstrated by H&E staining (Fig. 2). Cells within the defect region displayed a rounded morphology and embedded in lacunae (H&E staining) with typical morphology of chondrocytes. Besides, a glycosaminoglycans (GAGs)-rich extracellular matrix was found surrounding those round cells as demonstrated by TB (Fig. 3) and Saf.O (Fig. 4) staining. Furthermore, a Col II (IHC, Fig. 5) positively stained region was matched with those in TB and Saf.O staining. All evidences indicated that hyaline-like cartilage had formed at the damage region of the BMSCs-assisted Col I gel group at the first month. After 3 months, the damage region of the BMSCs-assisted Col I gel group still displayed a hyaline-like cartilage formation with spherical cells residing within lacunae (H&E staining) and matrix positive staining for GAGs (TB and Saf.O staining) and Col II (Col II IHC staining). What is more, the thickness of neo-cartilage tissue decreased and was similar to adjacent normal tissue. Six months later, the round chondrocytes in the damage region of the BMSCs-assisted Col I gel group still secreted large amount of cartilage-specific matrix (TB, Saf.O and Col II IHC staining) and the thickness of the regenerated cartilage region was gradually returned to consistent with neighboring healthy cartilage tissue. The prolonged and intense secretion of cartilage-associated matrix confirmed that the new cartilage tissue in BMSCs-assisted Col I gel group could be long-term maintenance.

In contrast, the defect sites of the UT group had distinct cracks and the structure of neo-tissue was predominantly fibrocartilage. Only few round cells with typical chondrocyte phenotype (H&E staining) and little amount of cartilage matrix formed (TB, Saf.O and Col II IHC staining). The treatment method of UT group was similar to marrow stimulation techniques through recruitment of endogenous MSCs from subchondral bone and formation blood clots repairing cartilage damage. However, because of the absence of scaffolds or other factors to regulate cell differentiation and promote the secretion of structurally consistent ECM, coupled with the inherent predisposition of chondrocytes to dedifferentiate nature, the treatment technique would result in fibrocartilage formation with inferior quality, and long-term studies confirmed that this method did not persist [2, 4, 5]. This view was confirmed in our study. Three months later, the defect site of the UT group basically filled with neo-tissue, the regenerated tissue was predominantly fibrous-like cartilage. There was no representative chondrocytes and the cartilage matrix GAGs and Col II existent (HE, TB, Saf.O and Col II IHC staining). The repair situation was significant deterioration compared to the first month. After 6 months, the defect site of UT group was also filled with amorphous fibroblast-like cells and fibrous tissue and had obvious surface depressed appearance.

**Scheme 1:**
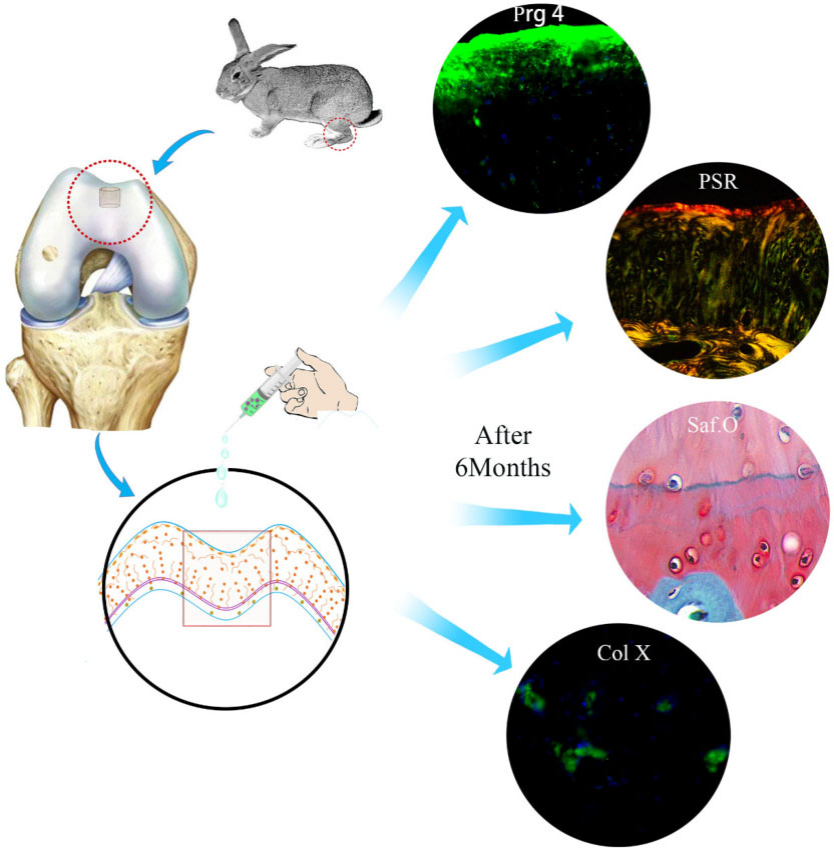
Schematic illustration of the experimental design and results

A quantitative scoring for the histological assessment was further performed (Fig. 2C). It would be evaluated from different parameters, including cartilage morphology, thickness, regularity, chondrocytes clustering or not, neo-cartilage GAGs content, and adjacent GAG and cell content. At the first month, there was no statistically significant difference between the UT group and the BMSCs-assisted Col I gel group. By 3 and 6 months later, the Col I gel group showed higher histological scores compared to UT group. By 6 months later, the histological scores of the Col I gel group was infinitely close to the maximum score (max score = 19), indicated the morphology, thickness, cell arrangement and matrix formation of the neo-tissue were similar to normal cartilage tissue (Scheme 1).

**Figure 6:**
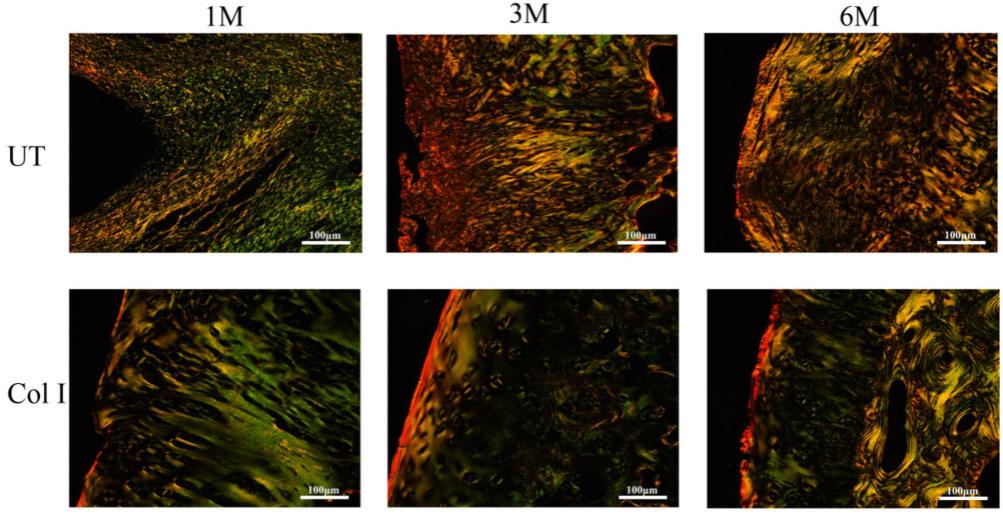
The PSR staining of the regenerated tissue at 1, 3 and 6 months. The scale bar is 100 μm

In PSR-stained sections (Fig. 6), the weak birefringence with yellowish green color represented Col II and strong birefringence with thin reddish-yellow color represented Col I. In UT group, the collagen fibers were mainly composed by Col II at 1 month, but the arrangement of fibers was irregular. With increase in time, the Col II in UT group was gradually replaced by Col I and the orientation of fibers was still random. In contrast, in BMSCs-assisted Col I gel group, Col II regular distributed in the cartilage repair site. Col I only accumulated in the superficial zone and oriented parallel to the surface of cartilage. After 3 months, the cartilage repair sites of the BMSCs-assisted Col I gel group were also mainly composed by Col II except the superficial region containing Col I. More importantly, the arrangement of collagen fiber became more regular and similar to normal cartilage. It was roughly divided into three distinct zones: the Col I in the superficial layer was oriented parallel with the surface, and the Col II of the deep layer was orientated perpendicular to the surface anchoring to the subchondral bone plate, but the intermediate layer Col II situated in transition between superficial and deep zone. By 6 months later, there was no Col I formation in cartilage zone except the superficial region and the collagen fiber arrangement was more regular and distinct.

**Figure 7:**
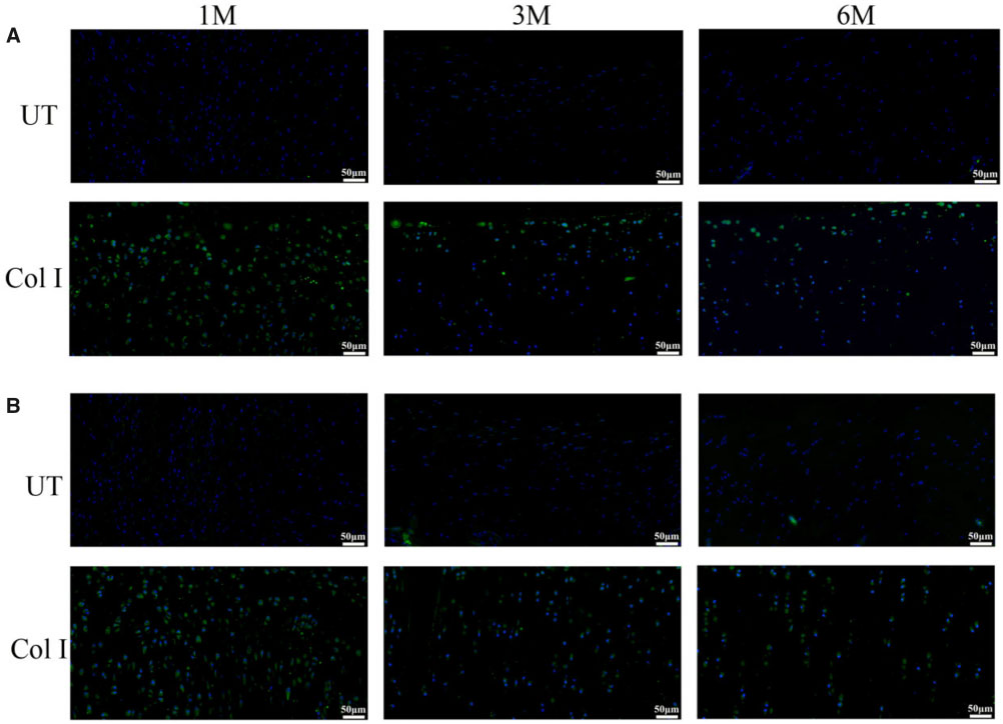
The immunofluorescence staining for BMP-2 (**A**) and TGF-β1 (**B**) of the regenerated tissue in UT group and BMSCs-assisted Col I group at 1, 3 and 6 months. The scale bar is 50 μm

The content of growth factors which related to the chondrogenic differentiation of BMSCs was detected by IF staining (Fig. 7). TGF-β1 and BMP-2 were existed around the cells in the regenerated region of BMSCs-assisted Col I hydrogel group. Then the content of TGF-β1 and BMP-2 significantly decreased with the endochondral ossification process and the decrease of cells. Due to the absence of scaffolds and BMSCs, there were no TGF-β1 and BMP-2 expression which was potent signals for initiating the chondrogenesis, it might be one of the reasons resulting in the poor repair effect in the UT group. This result demonstrated to some extent that the implantable BMSCs could promote the regeneration of cartilage. In a word, Col I gel could effectively induce the chondrogenic differentiation of exogenous BMSCs, promote cartilage repair and maintain for long term.

### Functional cartilage region and homeostasis restoration in BMSCs-assisted Col I gel group 

#### Calcified cartilage and tidemark formation

**Figure 8:**
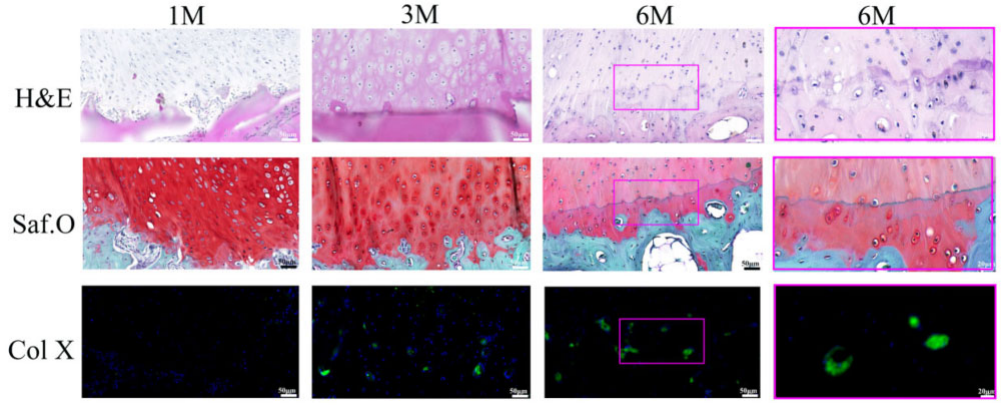
The regenerated interface zone of the BMSCs-assisted Col I group at 1, 3 and 6 months. The scale bar on the left three columns are 50 μm, and the right column is 20 μm

After 1 month implantation, there were basically no hypertrophic chondrocytes (H&E and Saf.O staining) and Col X (Col X IF staining) formation at the interface zone between neo-cartilage and subchonral bone (Fig. 8). From 1 to 3 months, chondrocytes at the interface region had hypertrophied morphology and expressed Col X (H&E, Saf.O and Col X IF staining). A spontaneous endochondral ossification process initiated, resulting thinning of neo-cartilage and being replacing by subchonral bone tissue. Six months later, the cartilage thickness in the BMSCs-assisted Col I gel group was nearly the same with the adjacent normal cartilage. Between the highly vascularized subchondrol bone layer and avascular cartilage layer, a distinct zone containing cartilage matrix and Col X (H&E, Saf.O and Col X IF staining) which was indicative of calcified cartilage was formed. The calcified zone played an important role in preventing the vascularization of cartilage zone and maintaining long-term stability of the neo-cartilage tissue [13, 14]. Most notably, a continuous strong hematoxylin-stained neo-tidemark with representative trilaminate appearance was gradually developing [35]. The tidemark represented a calcification front and it would replicate with the calcification front advancing into the non-calcified cartilage zone [35, 36]. After 6 months, a normal trilaminate tidemark kept with no duplication appearance, indicating no bony invasion past the calcified cartilage layer into the regenerated cartilage layer and no cartilage degeneration phenomenon.

#### Superficial cartilage formation

**Figure 9:**
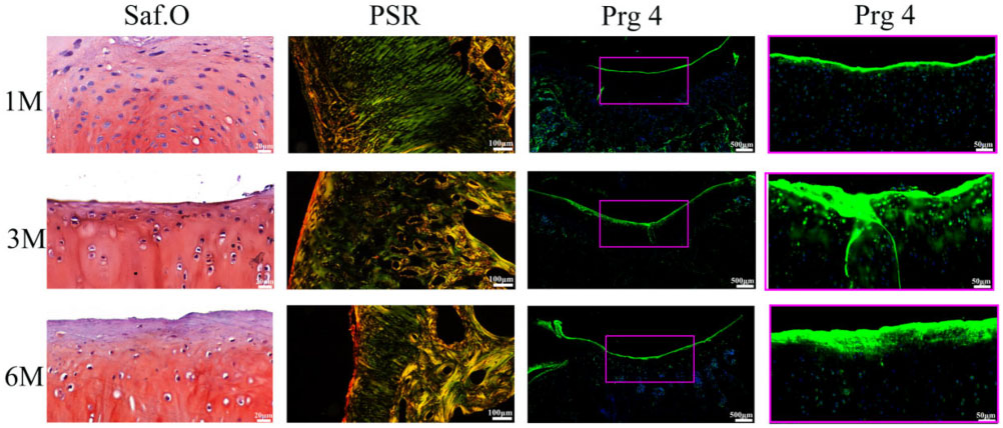
The regenerated superficial zone of the BMSCs-assisted Col I group at 1, 3 and 6 months. The scale bar of Saf.O staining is 20 μm, and the PSR staining is 100 μm. The scale bar of the Prg 4 IF staining on the left column is 500 μm, and the right column is 50 μm

The superficial region in normal cartilage contained flat chondrocytes, Col I fibers which oriented parallel to the surface of cartilage and specially expressed Prg 4 with lubricating function [6, 7]. After 1 month implantation, a few chondrocytes (H&E and Saf.O staining) became flat appearance. Cells (H&E and Saf.O staining) and Col I fibers (PSR, Fig. 6) lined up parallel to the cartilage surface at the superficial zone, while there was Prg 4 (Prg 4 IF staining) expressing in the region (Fig. 9). It is demonstrated that the impotent superficial structural with lubricating function had regenerated at the first month. It was worth noting that the superficial cartilage formed and the secretion of Prg 4 appeared in the initial stage, before the chondrocytes hypertrophy and endochondral ossification process. Some studies had confirmed that Prg 4 was an important regulator in skeletal development and homeostasis supporting in the mature skeleton [9, 10]. What is more, Prg 4 protected cartilage, prevented chondrocytes apoptosis, cartilage deterioration and inhibited the process of osteoarthritis by providing boundary lubrication, reducing friction and inhibiting the transcriptional programs that promote cartilage catabolism and hypertrophy [11, 12]. Therefore, the superficial region formation in the initial stage might be a key role for regulating calcified cartilage regeneration and maintaining long-term stability. With the increasing of implantation time, the expression of Prg 4 maintained throughout the entire repair process, the thickness of the superficial region and the fluorescence degree of Prg 4 had markedly increased.

#### Cartilage homeostasis construction

Some researchers defined the cartilage homeostasis as a complete tissue structure with corresponding functions and without structural or cellular damage [6, 7]. The cartilage could be divided into three distinct zones basing on different anatomical structures and functions. It has been confirmed that the superficial and calcified cartilage had respectively regenerated at 1 and 6 months. The intermediate zone contained round chondrocytes and produced abundant cartilage matrix to support the biomechanics for cartilage functions [7, 8]. This region also regenerated at the first month and maintained throughout the entire repair process. In addition, there was absence the phenomenon of cartilage clefts, chondrocytes cloning, loss of metachromasia, duplication of the tidemark and other degenerative changes in BMSCs-assisted Col I gel group at 6 months, confirming the new homeostasis has been reconstructed and effectively treatment might be maintained in long term [6, 7, 37].

## Conclusions

In this study, tissue-induced injectable Col I hydrogel was chosen as scaffold to overcome the limitation that the clinical treatment methods for cartilage defect repair. The results demonstrated that the BMSCs-assisted Col I gel could promoted regeneration of cartilage defect with complete anatomical structure and corresponding functions. Especially, the remodeling of the superficial cartilage with lubrication of Prg 4 and the calcified cartilage with barrier function between avascular cartilage and hypervascular subchondral bone played an important role in maintaining cartilage homeostasis and inhibiting the process of regenerated tissue degeneration. Therefore, BMSCs-assisted Col I gel for cartilage defect repair might have tremendous developing potentiality and clinical application value for maintaining long-term stability and avoiding the recurrence of postoperative arthritis.

*Conflict of interest statement*. None declared.
